# Bone remodeling around implants placed after socket preservation: a 10-year retrospective radiological study

**DOI:** 10.1186/s40729-021-00354-7

**Published:** 2021-07-29

**Authors:** Crespi Roberto, Toti Paolo, Crespi Giovanni, Covani Ugo, Brevi Bruno, Menchini-Fabris Giovanni-Battista

**Affiliations:** 1 School of Dentistry, Saint Camillus International University of Health and Medical Sciences, Via di Sant’Alessandro, 8, 00131 Rome, Italy; 2 Department of Stomatology, Tuscan Stomatological Institute, c/o Versilia General Hospital, via Aurelia 335, 55049 |Lido di Camaiore, Italy; 3 Department of Maxillo-Facial Surgery, Hospital and University of Pisa, via Paradisa 2, Pisa, 56124 Italy

**Keywords:** Alveolar ridge preservation, Split crest procedure, Xenogeneic bone substitute, Collagen sponge, Dental implants

## Abstract

**Background:**

To evaluate and compare the long-term clinical and radiological outcomes of post-extraction sockets after ridge preservation either with porcine xenograft or collagen alone. Patients underwent single-tooth extraction in the posterior mandible. Fresh extraction sockets were filled with pre-hydrated cortico-cancellous porcine bone or collagen sponge. Two or 3 months later, a ridge expansion technique with immediate implant positioning placement was performed. Primary (alveolar width changes) and secondary outcomes (adverse events and long-term maintenance of buccal plate covering the implant) were evaluated.

**Results:**

Thirty-four women and 20 men were selected: 30 implants (group A) placed into healed post-extraction sockets grafted with porcine bone and 24 (group B) into sockets filled with a collagen sponge. There was a significant loss in width in both groups from the first and second surgery (ranging between 2.7 mm and 4.5 mm). The ridge splitting with bone expansion resulted in significant long-term increases in width for both procedures and implant sites. Non-significant differences in alveolar width were registered between the groups at 10-year follow-up even if the analysis of the implant buccal bone coverage suggested that group A had significantly worst results.

**Conclusions:**

Porcine bone group had significantly better short-term outcomes with lower long-term maintenance of the buccal plate.

## Background

Considerable difficulties in positioning dental implants in fresh extraction sockets could be associated with gradual loss of height of the alveolar walls or damage of the buccal bone plate, especially in the anterior maxilla region where the maintenance of sufficient bone volume allowed for achieving the best results in terms of biological and aesthetic outcomes [1–4]. It is therefore not surprising that several surgical procedures, such as guided bone regeneration [5, 6], or grafting augmentation procedures with or without autologous bone which could be substituted with any bone replacing material (such as allogeneic, xenogeneic, or synthetic bone substitutes) [7–9] were recommended to maintain the volume of the alveolar process during the healing phase.

Due to their excellent biocompatibility and bioactivity, anorganic animal bone particles were used as graft materials for both the ridge preservation and the maxillary sinus augmentation, so providing sufficient gain to achieve adequate bone volume and quality [10–13]. Nevertheless, some synthetic materials were tested, the porcine bone, used for socket filling after tooth extraction, seemed to behave in a similarly in the histological pattern and bone remodeling process [14, 15]. Meanwhile, it was assessed the advance in increasing the bone mineral content of the buccal bone defects after filling with collagen alone [16].

## Methods

The present study aimed to compare and evaluate the clinical and radiological outcomes of delayed implant placement in healed extraction sockets previously grafted with cortico-cancellous porcine bone versus sockets filled by collagen alone. The study reported long-term findings at 10 years.

### Patient selection

In a retrospective analysis, all subjects were selected among a cohort of patients who were consecutively treated between January 2008 and June 2010 at Tuscan Stomatological Institute and followed up at the Complex Operating Unit of Maxillo-Facial Surgery of the University of Pisa. This study followed the Declaration of Helsinki on medical protocol and ethics, and the regional Ethical Review Board of the University of Pisa approved the present analysis.

**Fig. 1:**
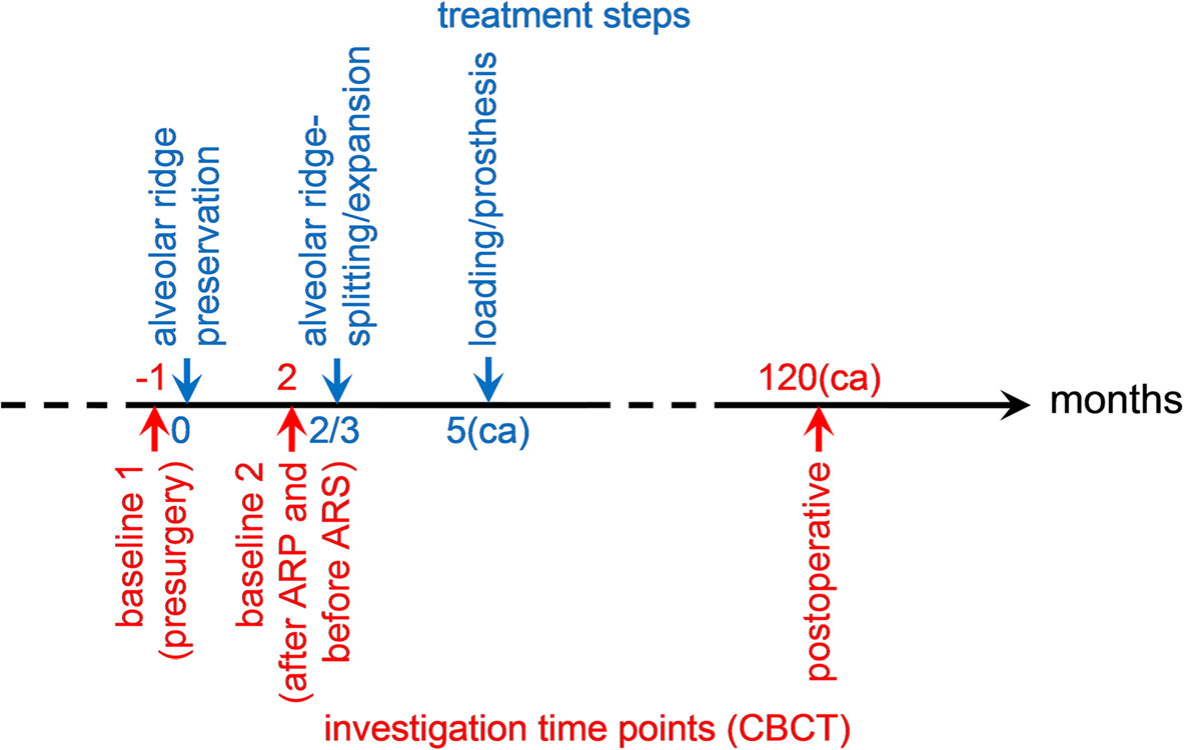
Scheme for chronology of surgical interventions and of investigations at different points in time

Only patients matching the following criteria were included in the further data analysis: patients who signed an informed consent form (18 years old or older); single tooth extraction in the posterior area; alveolar ridge/socket preservation (ARP) in posterior extraction sites with either a cortico-cancellous porcine bone or a collagen sponge; delayed alveolar ridge-splitting/expansion (ARS) technique with immediate dental implant placement; report of a clinical follow-up period up to 10 years from the first surgery; preoperative (baseline 1) and postoperative (baseline 2 and 10 years) mandibular computerized tomography scans as well as displayed in the diagram (Fig. 1) describing the chronology of surgical interventions and of investigations at different points in time.

Patients were excluded if any of the following items were shown in the medical records: history of chronic and systemic diseases coming out during follow-up that contraindicate oral surgery; long-term non-steroidal anti-inflammatory drug therapy; oral or intravenous administration of bisphosphonate drugs; heavy smokers with a cigarette consumption higher than 10 cigarettes/day.

### Surgery

One hour before surgery, each patient received prophylactic therapy (1 g of amoxicillin or clindamycin 600 mg if allergic to penicillins, then 1 g amoxicillin or 300 g clindamycin twice daily for 5 days. All patients were treated under local anesthesia using optocaine 20 mg/mL with adrenaline 1:100,000. According to the basic steps of the “socket-plug” technique teeth were extracted without flap elevation to maximally preserve the hard and soft tissues, avoiding periosteum detachment and traumatic tooth extraction.

**Fig. 2:**
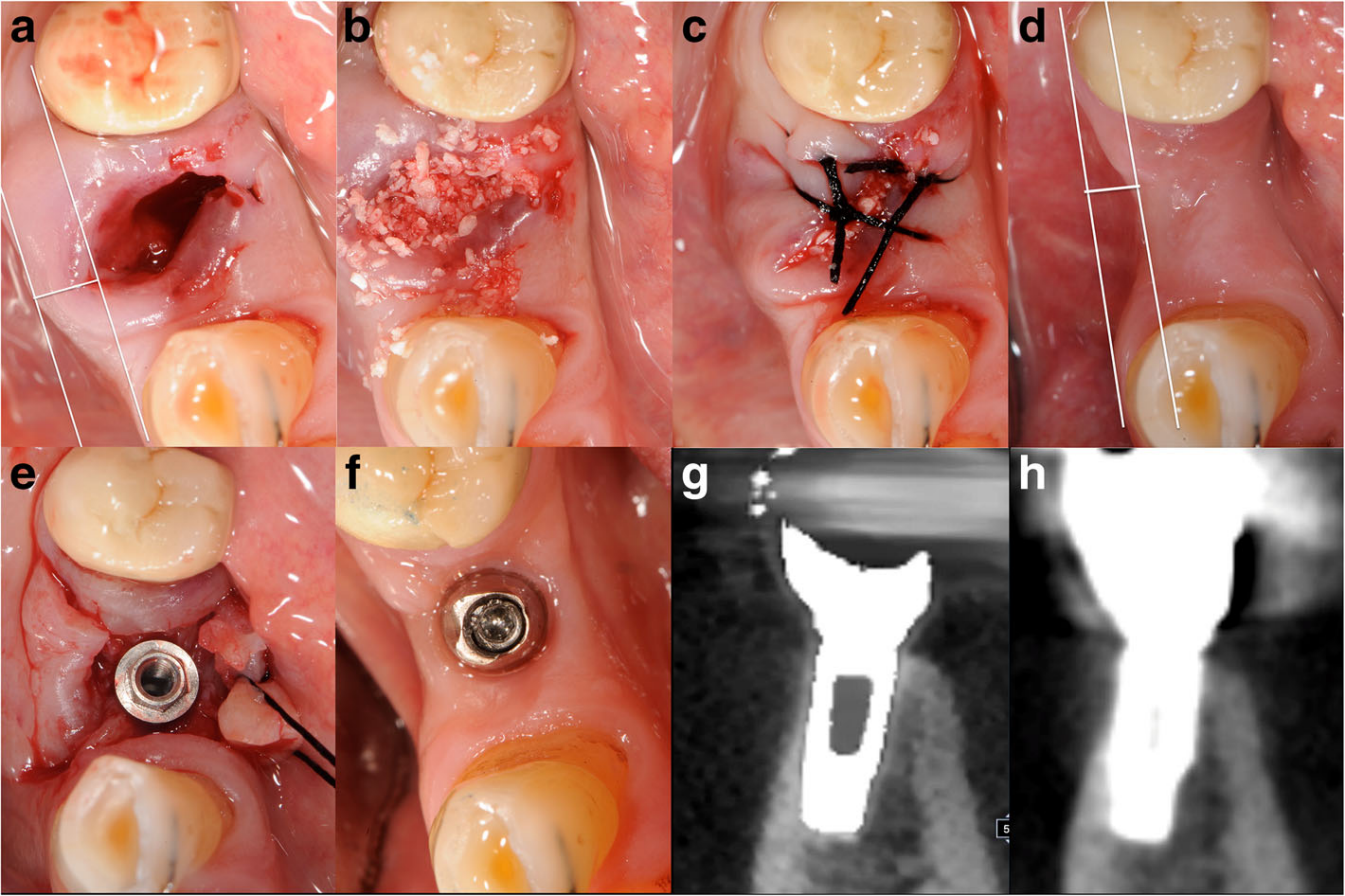
Clinical photographs. **a** fresh socket; **b** cortico-cancellous porcine and filled socket; **c** collagen sheet covering secured with silk sutures; **d** site healing at 3 months; **e** implant placement into healed site; **f** healed site; **g** cone beam computed tomography 3 years after implant placement and **h** after 10 years from first surgery1

In group A, extraction sockets were grafted up to the buccal and palatal margin of the alveolar wall with a pre-hydrated cortico-cancellous porcine bone (particle size between 600 and 1000 μm, MP3, OsteoBiol®, Tecnoss®, Coazze, Italy). Subsequently, a collagen sheet (Condress®, Abiogen Pharma, Pisa, Italy) was placed to cover the socket, and secured with silk sutures [14] to stabilize blood clots and to prevent the leakage of graft particles (Fig. 2a–c).

**Fig. 3:**
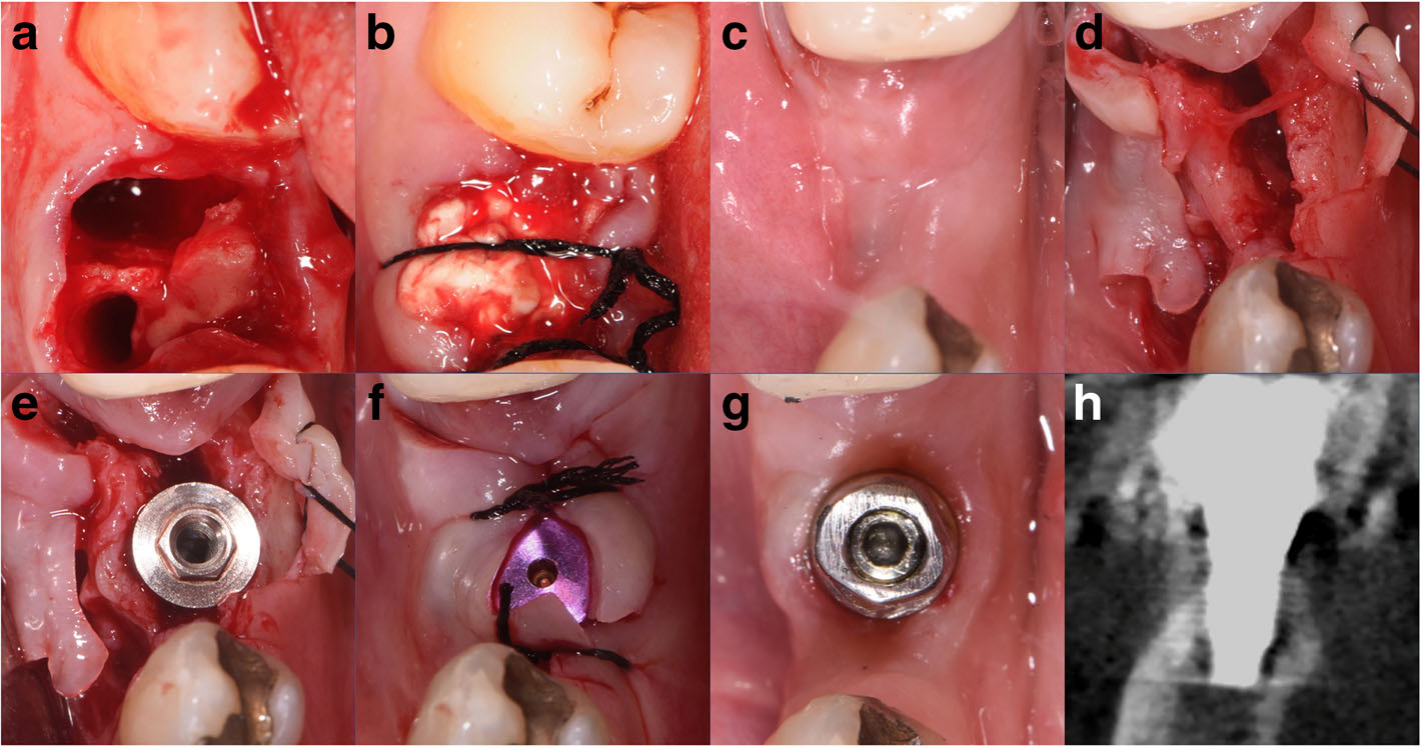
Clinical photographs: **a** fresh socket; **b** collagen filling the socket; **c** site healing at 3 months; **d** site after split crest; **e** implant placed; **f** site secured with silk sutures; **g** healed site; **h** cone beam computed tomography after 10 years from first surgery

In group B, the extraction sockets were filled with collagen sponges. If sutures were placed, they stabilized the collagen [16]. The sutures were not tight without primary closure of the wound [16]. The wound was left to heal by secondary intention (Fig. 3a-c).

In both groups 8–10 weeks after surgery (Fig. 2d; Fig. 3d), an alveolar ridge expansion was performed by an electromagnetic device (Magnetic Mallet, www.osseotouch.com, Turbigo, Milano, Italy) [17]. A palatal or lingual incision in crestal direction was performed followed by two transperiosteal incisions made perpendicular to the initial incision on either side allowing the raising of a partial-thickness flap. After the flap reflection, two vertical grooves were made by the penetration of the vestibular cortical bone plate one on the mesial aspect and one on the distal aspect of the flap edges by keeping a safe distance of 1 mm from the adjacent teeth (Fig. 2e). A blade directly attached to the electromagnetic device performed the crestal bone incision maintaining a zone of spongy bone beneath the cortical plate with a minimum thickness of 1.5 mm and penetrated the alveolar ridge from 7 to 11 mm deep. The bony wall was slowly expanded and the facial bony plate was dislocated in a buccal direction with a progressive series of bone expanders attached to the handpiece of the electromagnetic device.

The recipient site was prepared undersized by 1 mm than the implant diameter in the newly created space obtained by expanding the bone tissue both laterally, against the preexisting lateral walls, and apically, moving up and compressing it. Titanium Plasma Spray implants with a machined neck for 0.8 mm, and a rough surface, body with a progressive thread design (Seven, Sweden and Martina, Padua, Italy), were firmly seated with native bone engagement (Fig. 2e; Fig. 3e, f).

The buccal flap was apically repositioned, sutured to the margin of the palatal/lingual flap, and anchored with a loose loop to the periosteum at the level of the alveolar mucosa. The surgical field was covered by collagen that was inserted under the undermined keratinized mucosa that lined the flap edges. The collagen ensured that the bleeding stops and intended to stabilize the blood clot [18].

### Prosthetic protocol

After 2 months of submerged healing, dental implants were loaded (Fig. 2f; Fig. 3g) with two different polyvinylsiloxanes impression materials (Flexitime Heavy plus Flow, Heraeus/Kulzer, Milan, Italy) with an individual acrylic impression tray. The final crown restoration was applied onto custom abutment. After a temporary crown restoration, definitive ceramic fused-to-metal restoration was fabricated 5 months after the first surgery (Fig. 2g, h; Fig. 3h).

### Primary predictors

Group A: pre-hydrated cortico-cancellous porcine bone

Group B: collagen sponge

### Secondary predictors

Patient gender and implant site

### Radiographic examination and outcome variables

Computerized tomographic scans were acquired with a device dedicated to dental and maxillofacial imaging (Gendex GXCB-500; Gendex Dental Systems Hatfield, PA, USA) with the following setting: 120 kV, 30.89 mAs, 200 μm isotropic voxel, 8.5 cm field of view (FOV). The CBCT scans of each patient were then transferred to a single-blind examiner radiologist for evaluation which performed all the measurements.

Preoperative and postoperative scans were modified appearing to be superimposable according to Crespi and co-workers [19]. CBCT cross-sectional images were extrapolated from each set of the three scans superimposed in the space (using a triangulation of point data: two spines of Spix and midpoint of the segment connecting the two mental foramina). Measurement of the alveolar width (AW) was performed in a cross-sectional image that passed through the long axis of the implant and along a line perpendicular to it passing between the most coronal points of the palatal bone level and the most prominent point of the buccal bone. The position of the implant served as the point of reference for the preoperative superimposed measurements. The loss in bone width (ΔAW) was given by Equation 1: ∆AW = AW_postop_ − AW_preop_ (Eq. 1)

Maintenance of the buccal plate was measured on the above-mentioned cross-sectional image at 10 years of follow-up; it was assessed with a dichotomic value (yes/no).

In particular, the blind measurer relied on the subjective criterium such as “cutoff of at least 70% or more percentage of bone/implant coverage at the buccal side.”

## Statistical analysis

A specific program dedicated to statistics (Statistics Toolbox, MATLAB 7.11; The MathWorks) was employed for all the analyses. Normal distributions of groups and subgroups were not confirmed, so sample description, dispersion, and analysis used a nonparametric approach. Bone width values were reported as a median and interquartile range, $\overset{\sim }{m}$(iqr) and rounded to the nearest decimal.

The Null hypothesis: H0, there was no difference when healed posterior mandibular site underwent alveolar ridge preservation with socket-plug technique (pre-hydrated cortico-cancellous porcine bone substitute versus collagen sponge alone) was treated by ridge splitting and simultaneous implant placement.

Friedman’s test has been employed as a non-parametric analysis of variance test (ANOVA). To compare the differences between radiographic values between groups at every time point, post-hoc pair-wise comparisons were performed by Mann–Whitney tests for independent samples. Differences between times were evaluated with the Wilcoxon signed-rank test. The significance was set at a level of 0.01.

## Results

### Clinical outcomes

Fifty-four subjects, 34 women and 20 men (with a mean age of 53.8 ± 7.1 years with a range from 41.8 to 69.1 years) were selected. A single tooth per patient was analyzed, thus bringing the number of dental implants with delayed placement to 54:30 implants in group A were placed in preserved post-extraction sockets grafted with a porcine bone; 24 implants belonging to group B were placed in preserved post-extraction sockets filled with a collagen sponge alone. During the healing period, neither soft tissue infection episodes nor signs of exposed bone were mentioned in the case sheets attesting to the achievement of complete wound healing around the temporary restoration.

### Radiographic evaluation

Absolute values of the alveolar bone widths (AWs) and their changes in time (ΔAW) were shown in Table 1. The site of the implant, i.e., bicuspid versus molar, had been checked to test the influence of the variance in the alveolar width change between the two groups with regard to this confounding factor. On the other side, the Friedman tests did not reveal any significant influence on the changes between the genders (Table 1).

**Table 1:** Median and interquartile range $\overset{\sim }{m}$(iqr) of the alveolar bone width at baseline 1 (pre-extraction) and baseline 2 (before delayed dental implant placement) and after 10 years for sites with porcine bone graft (A) and collagen sponge (B)

		Alveolar width (AW)			Change at the alveolar ridge (ΔAW)			Intragroup analysis (*p* value)		
Group	*N*°	bsl 1	bsl 2	10 years	bsl 1→bsl 2	bsl 2→10 years	bsl 1→10 years	bsl 1 vs bsl 2	bsl 2 vs 10 years	bsl 1 vs 10 years
	Overall sites									
A	30	10.9(2.3)	8.4(2.1)	9.4(1.6)	− 2.7(0.9)	+ 1.3(1.1)	− 1.3(1.6)	**< 0.0001**	**< 0.0001**	**< 0.0001**
B	24	10.6(2.0)	7.1(1.1)	9.0(1.5)	− 3.9(1.4)	+ 1.8(1.1)	− 2.2(1.6)	**< 0.0001**	**< 0.0001**	**< 0.0001**
Intergroup analysis (*p* value*)		0.8616	**0.0032**	0.1296	**< 0.0001**	0.0688	0.0863			
Effect of site (*p* value^)		**< 0.0001**	**< 0.0001**	**0.0099**	0.0176	0.6093	**0.0086**			
Effect of gender (*p* value^)		0.0442	0.0102	0.0448	0.7096	0.8234	0.8236			
	Premolar									
A	16	9.8(0.7)	7.1(1.3)	9.1(1.4)	− 2.8(1.0)	+ 1.5(1.5)	− 1.3(1.7)	**0.0004**	**0.0004**	**0.0004**
B	13	10.2(0.8)	6.7(0.7)	8.3(1.4)	− 3.3(1.1)	+ 1.7(1.4)	− 1.5(1.5)	**0.0002**	**0.0002**	**0.0002**
Intergroup analysis (*p* value*)		0.5524	0.1349	0.2816	0.0562	0.8434	0.2190			
	Molar									
A	14	12.1(0.3)	9.1(0.9)	9.9(1.8)	− 2.7(0.5)	+ 1.0(1.7)	− 1.8(2.0)	**0.0001**	**0.0001**	**0.0234**
B	11	12.1(0.6)	7.5(1.6)	9.4(1.2)	− 4.6(0.6)	+ 1.9(0.7)	− 2.7(1.1)	**0.0009**	**0.0009**	**0.0009**
Intergroup analysis (*p* value*)		1	**0.0005**	0.2743	**< 0.0001**	**0.0061**	0.2972			
	Premolar vs molar									
A (*p* value*)		**< 0.0001**	**< 0.0001**	0.0209	0.06772	0.0612	0.0843			
B (*p* value*)		**< 0.0001**	0.0594	0.0395	**0.0003**	0.3102	0.0137			

Results with significance in bold

^Non-parametric analysis of variance test (ANOVA Friedman test)

*Mann–Whitney tests

°Wilcoxon signed-rank test

The intragroup analyses suggested that significant width reductions of the alveolar processes were observed in both groups from baseline 1 to baseline 2, that is about 2/3 months after the alveolar ridge preservation and before the dental implant placement, with a loss ranging between − 2.7 and − 4.6 mm. This proved that, at least in the molar region, the alveolar ridge preservation technique with low absorption material should fulfill its purposes exactly matching the expectations of the clinician.

The radiological outcome of the alveolar ridge expansion procedure, measured from baseline 2 to 10 years, showed a significant increase in the alveolar width in both groups (A and B) and sites (bicuspid and molar); in fact, significant increases in width (ranging from + 1.0 mm to + 1.9 mm with *p* values ≤ 0.0009) were still visible after a decade.

Cross-group analyses suggested that loss in the alveolar width was higher in group B than in group A at least until the second surgery, i.e., alveolar ridge expansion (2/3 months after) for both implant sites.

Changes in alveolar width, albeit not significant in the premolar sites, ranged from 9.8(0.7) to 7.1(1.3) mm and from 10.2(0.8) to 6.7(0.7) mm for group A and B, respectively; on the contrary, they significantly changed from 12.1(0.3) to 9.1(0.9) mm and from 12.1(0.6) to 7.5(1.6) mm for group A and B, respectively, in the molar site.

Again, changes in the molar area indicated that alveolar ridge expansion led to an overall and statistically significant higher increase (*p* = 0.0061) in group B, + 1.9 (0.7 mm, than that reported in group A, + 1.0(1.7) mm. Furthermore, the remodeling process after tooth avulsion and before dental implant placement (2/3 months after ridge preservation) demonstrated that the premolar regions behaved significantly different (with *p* = 0.0003) when compared to the molar ones, even if for the extraction sockets filled with collagen sponges.

Finally, no significant differences were registered at 10-year follow-up between the two procedures and between the two implant sites (Table 1).

Overall radiologic outcomes (from baseline 1- to 10-year survey) calculated summing over time the changes in alveolar width after both surgeries (ARP and ARS) showed no significant differences between the group A and B.

The outcomes regarding the buccal contours demonstrated that few implants in group B (2 out of 24 placed in sockets preserved with collagen alone) had no complete buccal bone coverage, as visible in the CBCT images and segmentations. Moreover, the analysis of the distribution of the data (Table 2) demonstrated that group A showed significantly worst results over the long term than group B (Fisher’s exact test *p* value = 0.0005).

**Table 2:** Distribution about maintenance of the buccal bone plate at 10 years of follow-up for the porcine bone graft group (A) and collagen sponge group (B)

	Buccal bone maintenance	Buccal bone resorption	Fisher’s exact test
A	14	16	0.0005
B	22	2	

## Discussion

The present analysis aimed to understand how different bone substitutes with different rates of resorption (xenogeneic cortico-cancellous bone or collagen sponge) used in alveolar ridge preservation could affect long-term changes in alveolar width when socket preservation was combined with delayed alveolar ridge splitting/expansion technique and immediate implant placement.

As far as bone remodeling is concerned, the type of surgical procedure (traumatic/atraumatic) is one of the most active factors: the architecture of the tissues around the surgical site (hard and soft tissues) and dynamic of the healing process (wound closure and blood clot stabilization) were key drivers for success [20]. Among the several strategies used to prevent alveolar bone resorption, different methods of ridge preservation had been proposed, that range from atraumatic flapless tooth extraction aiming for undisturbed extraction wounds [21] to more complex and demanding socket-plug technique in combination with different grafting materials, barrier membranes, and additional surgical procedures [22].

Computerized tomography scans acquired immediately after extraction and then at 3 months after surgery revealed that sockets treated with porcine bone demonstrated a loss of less than 25% in width of the alveolar ridge. On the contrary, sockets filled with collagen sponge showed a significantly higher shrinkage dimension (about 35%) than that registered for group A. This could lead to the conclusion that the patients benefit from receiving grafting materials at the time of tooth extraction. Even if the present procedure seemed to be more demanding than the standard ones of the other authors, Jung and co-workers [23] attested that the xenogeneic bone substitutes appeared to be able to limit, up to a certain extent, the resorption of the alveolar process after tooth extraction; this was confirmed also in the present study where both groups (porcine bone and collagen alone) showed a dimensional shrinkage before the dental implant was placed. Furthermore, Ten Heggeler and co-workers [24] confirmed that alveolar ridge preservation utilizing the “socket-plug” technique may not prevent the physiological resorption of the alveolar bone, especially in molar areas. However, in the present study the healing pattern of extraction sockets preserved using collagen sponges seemed to have a behavior similar to that reported by Chen and co-workers [25], and by Amler [26]; the above-mentioned authors confirmed that two-thirds of the sockets appeared to be filled with the mineralized bone after only 40 days of healing.

The results achieved through the use of the collagen tablets [27] might explain the similar clinical outcomes reported in this study for the first 8–10 weeks in which socket healing moved through three fundamental phases: the first one—remodeling of the blood clot within a week after tooth extraction, the second—consistent deposition of temporary connective tissue within the first weeks of healing, and finally, laying down of the bony matrix and its mineralization in a less predictable time.

The ridge expansion technique led to an increase in the alveolar width that persisted to the last survey for both groups with a significantly higher augmentation in sockets filled with collagen than those grafted with porcine bone. Radiographic analysis of the collagen-grafted sites here reported, suggested that outcomes were similar to those described in previous studies, where new bone formation around implants placed in augmented bone in which reactive soft tissue was left in the defects had been evaluated using cone-beam computerized tomography (CBCT) [28, 29]. However, short-term cross-sectional images (not selected for the analysis) revealed that maintenance of buccal plate was similar in both groups; on the contrary, at the final check-up for radiologic evaluation differences were registered. Trend about the bone remodeling was confirmed by the maintenance of the buccal cortical plate around dental implants placed in the sites grafted with collagen (22 out of 24) but not in the areas treated with the porcine bone substitute; several cases of buccal cortical plate loss were registered.

A significant difference between the two groups might be explained by the steady-state bone remodeling activity of the peri-implant tissues which acted to balance the functional strengths and reaction of supporting tissues; so, a positive remodeling of the bone was a simple response to mechanical stress [30]. Bone substitute materials underwent volume resorption and complete replacement by new vital bone in a very long time (it could take more than 4 years); however, currently, from a clinical point of view, there was no evidence that the load-bearing capacity of augmented bone appeared to be different compared with the normal bone [31].

## Conclusions

The porcine bone group had significantly better short-term outcomes with lower long-term maintenance of the buccal cortical plate around dental implants. On the contrary, the collagen allowed the formation and preservation of the buccal cortical plate.

## Data Availability

The datasets used and/or analyzed during the current study are available from the corresponding author on reasonable request in STRUCT form with multiple strings/arrays running in MATLAB version 7.01 or later.

## Abbreviations

ARP: Alveolar ridge/socket preservation
ARS: Alveolar ridge-splitting/expansion
FOV: Field of view
AW: Alveolar width
ΔAW: Change in alveolar width
CBCT: Cone beam computerized tomography
